# Biosynthesis of lanosterol in *Escherichia coli*

**DOI:** 10.1016/j.synbio.2025.05.006

**Published:** 2025-05-13

**Authors:** Wenjie Sun, Yun Chen, Syed Bilal Shah, Yanfen Bai, Zaigao Tan

**Affiliations:** a State Key Laboratory of Microbial Metabolism, Shanghai Jiao Tong University, Shanghai, 200240, China; b Department of Bioengineering, School of Life Sciences and Biotechnology, Shanghai Jiao Tong University, Shanghai, 200240, China; c Shenzhen Institute of Advanced Technology, Chinese Academy of Sciences, Shenzhen, 518000, China

**Keywords:** Sterols, Lipids, Lanosterol biosynthesis, Tolerance

## Abstract

Lipid composition represents a significant differentiator across the three domains (eukaryotes, bacteria, and archaea) of cellular life. Eukaryotes possess distinct lipids, such as sterols and sphingolipids, generally, these are not commonly found in typical bacteria and archaea. Sterols play a pivotal role in eukaryotic cellular functions, lanosterol, a key precursor for animal and fungal steroids, has well established functions in eukaryotes, while its potential functions in bacteria remain largely uninvestigated. In this study, we genetically engineered *Escherichia coli* (*E. coli*) to reconstruct the biosynthesis of lanosterol, and successfully developed a novel *E. coli* strain capable of synthesizing lanosterol, although its specific location, such as whether it is incorporated into the cell membrane, remains to be further determined. Comprehensive characterization of the observed phenotypic changes has unveiled that, despite an unaltered growth rate under normal condition, the engineered *E. coli* strain displayed notably enhanced tolerance to various stresses. Subsequent analysis has indicated that lanosterol plays a role in preserving membrane integrity, fluidity, hydrophobicity, and ATP production, mirroring the functions of sterols in eukaryotes. This study unveils the unexpected capacity of *E. coli* to synthesize sterols, not only underscores the importance of lanosterol as a precursor for essential cellular lipids but also offers fresh insights into the potential functions of sterols within bacterial systems.

## 1 Introduction

Cellular life is traditionally classified into three domains: eukaryotes, bacteria, and archaea [1]. Lipids are major constituents of all living cells, playing a crucial role in various cellular processes, particularly, they form a bilayer that acts as a barrier separating the cell's interior from its exterior [2,3], as well as a matrix supporting the diverse functions of membrane proteins [2,3]. The composition of lipids is a key factor distinguishing eukaryotes from bacteria and archaea [4]. Although eukaryotes also possess bacterial-type phospholipids, they have unique lipids that are absent in both bacteria and archaea, such as sterols and sphingolipids [5]. This diversity in lipid composition, known as “the lipid divide”, is characterized by the presence of unique lipids in eukaryotes, such as sterols and sphingolipids, which are absent in both bacteria and archaea [6].

Sterols are tetracyclic triterpenoid lipids that are essential for critical cellular functions in all eukaryotes. They maintain membrane fluidity and integrity, enhance stress tolerance, modulate of ATPase activity, and facilitate cell signaling [7]. Lanosterol, a tetracyclic triterpenoid, is a key precursor for all animal and fungal steroids, which is converted to ergosterol in yeast and fungi, and to cholesterol in mammals [8,9]. Its biosynthesis is a critical step in the production of these essential sterols, highlighting its fundamental importance in cellular lipid metabolism. Despite the well-documented roles of sterols in eukaryotes, the functional potential of lanosterol and its derivatives in other biological systems remains underexplored. In bacteria, for example, the presence of sterols is rare, and the few known sterol-producing bacteria, such as aerobic methanotrophs, planctomycetes, and myxobacteria, are often challenging to culture and genetically manipulate [[10], [11], [12], [13], [14]]. This limits our ability to study the functional impact of sterol incorporation in bacteria. In contrast, model bacteria such as *Escherichia coli* (*E. coli*) offer unparalleled advantages for such studies. They possess genetic tractability, favorable growth conditions, well-characterized biochemistry and physiology, and versatile genetic manipulation tools [15,16], making them ideal candidates for exploring the functional impact of sterol incorporation in bacteria.

In this study, we successfully reconstituted the biosynthesis pathway of lanosterol in the model bacterium *E. coli* through genetic engineering. We enabled this bacterium to produce lanosterol, a crucial eukaryotic sterol precursor, this innovative approach allowed us to investigate the functional impact of lanosterol to bacterial. Our results demonstrate that the presence of lanosterol enhances the engineered *E. coli* to exhibit increased tolerance to adverse stresses. Further analysis revealed that lanosterol contributes to maintaining several critical membrane properties, including integrity, fluidity, and ATP production. This study not only highlights the importance of lanosterol as a precursor for essential cellular lipids but also provides new insights into the potential functional roles of sterols in bacterial systems.

## 2 Materials and methods

### 2.1 Strains and plasmids

All plasmids and strains used in this study are listed in Table S1. All strains are derivatives of *E. coli* MG1655 K-12. Gene screening was conducted by assessing protein soluble expression in *E. coli.* Candidate genes were cloned into the P1 site of the pCDF-duet 1 vector (Novagen) using the Seamless Cloning Kit (Beyotime) to yield the pCDF-*gene* plasmid. The resulting plasmids were introduced to *E. coli* strain BL21(DE3) respectively. Their solubility was evaluated under 0.1 mM IPTG induction.

For the lanosterol pathway construction, chromosomal editing was performed using CRISPR-Cas9 method [17]. The gene *NusA*, labeled with a solution-aid label, was cloned from pSJ7 vector, used for enhance the solubility of LSS. The M1-12-*NusA*-*lss* cassette was inserted at the *ldhA* site. The native promoter of the *dxs* gene was replaced with the M1-37 promoter using one-step recombination (FLP-FRT) [18]. When necessary for the strain construction, antibiotics were added at the final concentrations of: ampicillin (100 μg mL^−1^), spectinomycin (50 μg mL^−1^) and kanamycin (50 μg mL^−1^).

The candidate gene *lss* from *Methylococcus capsulatus* were synthesized by AZENTA Life Biotech with codon optimization, and then using Phanta Max Super-Fidelity DNA polymerase (vazyme) for vector construction cloing.

### 2.2 Growth conditions and characterization

All tolerance experiments were conducted using a clear-bottom 96-well plate containing 200 μL of MOPS minimal medium (40 mM MOPS, 4 mM tricine, 0.01 mM FeSO_4_, 9.5 mM NH_4_Cl, 0.276 mM K_2_SO_4_, 0.5 μM CaCl_2_, 0.525 mM MgCl_2_, 50 mM NaCl, 0.292 nM (NH_4_)_2_MoO_4_, 40 nM H_3_BO_3_, 3.02 nM CoCl_2_, 0.962 nM CuSO_4_, 8.08 nM MnCl_2_, 0.974 nM ZnSO_4_, and 1.32 mM K_2_PO_4_) with 2 % (wt/v) glucose medium and inhibitors of specific concentration in a clear-bottom 96-well plate [19]. The experiments were carried out at 37 °C with an initial pH of 7.0. High temperature tolerance was assessed at 42 °C. The specific growth rate (μ, h^−1^) was calculated by fitting the equation OD_550,t_ = OD_550,0_ e^μt^ to the exponential growth phase. All estimated μ values had an R2 value of at least 0.95.

### 2.3 Extraction and detection of sterols

Sterols were extracted using a modified Christoph Müller method [20]. In brief, 40 mg of mid-log phase *E. coli* cells were harvested, washed twice with PBS buffer, and then mixed with 1 mL of 2 M sodium hydroxide (NaOH). The mixture was heated at 70 °C for 1 h to undergo saponification. After incubation, 1 mL of the lysed cell suspension was transferred to a new tube. Subsequently, 650 μL of methyl-tert-butylether (MtBE) and 100 μL of internal standard (5α-cholestane in MtBE, 10 μg mL^−1^) were added. The mixture was vigorously vortexed for 30 min, followed by centrifugation, and the upper organic layer was transferred to a fresh tube. The lysed cell suspension was subjected to another extraction step with an additional 750 μL of MtBE. The initial and second organic layers were combined and dried under nitrogen. Then, 170 μL of MtBE was added to dissolve the dried residue. Afterward, 20 μL of cholesterol standard (10 μg mL^−1^) and 10 μL of MSTFA/TSIM (9:1) (a mixture of N-methyl-N-trimethylsilyltrifluoroacetamide (MSTFA) and 10 % (vol/vol) N-trimethylsilylimidazole (TSIM)) were added to the sample. The sample was incubated at 22 °C for at least 0.5 h before being analyzed by GC-MS [20].

### 2.4 Membrane and cell surface characterization

Membrane integrity was assessed using SYTOX green (Invitrogen) staining [21], while membrane fluidity was evaluated using 1,6-diphenyl-1,3,5-hexatriene (DPH) (Invitrogen) [22]. The extraction of membrane-bound fatty acids was performed using a modified Bligh and Dyer method [23], followed by analysis using gas chromatography-mass spectrometry (GC-MS) [22]. To extract phospholipids, 1 mL of cell suspension was mixed with 3.75 mL of a chloroform/methanol/12 N HCl solution (2/4/0.1, v/v). The mixture was thoroughly mixed, and then 1.25 mL of chloroform was added with vortexing for 30 s, followed by the addition of 1.25 mL of water with similar mixing. After centrifugation at a low speed for 10 min, the lower chloroform layer was carefully removed and transferred to a glass tube for evaporation. Subsequently, 0.2 mL of chloroform/methanol (2/1, v/v) was used to resuspend the resulting pellet. The phospholipids obtained were subjected to PE/PG/CL content analysis using the corresponding detection assay kits (Abcam) according to the manufacturer's protocol.

### 2.5 Extraction and esterification of *E. coli* membrane-bound fatty acids

Membrane-bound fatty acids were extracted using a modified Bligh and Dyer method [23], and analyzed by gas chromatography-mass spectrometry (GC-MS) [22]. To briefly summarize the procedure, mid-log cells were first centrifuged and washed twice with cold double-distilled water (ddH_2_O). The resulting pellet was then resuspended in 1.4 mL ddH_2_O, and 10 μL of a 1 μg/μL pentadecanoic acid (C15:0) solution (dissolved in ethanol) was added as an internal standard. Next, 1.4 mL of methanol was added to the cell suspension and transferred to a new glass tube. The samples were sonicated for 10 min, followed by incubation at 70 °C for 15 min and centrifugation at 5000×*g* for 5 min. The supernatant was carefully transferred to a new glass tube, while the cell pellet was resuspended in 0.75 mL of chloroform and shaken at 37 °C, 150 rpm for 5 min. The supernatant and pellet suspension were combined, vortexed for 1 min, and centrifuged at 5000×*g* for 2 min. The bottom phase was transferred to a new glass tube and dried under nitrogen gas. Subsequently, a mixture of 2 mL methanol:sulfuric acid (98:2 v/v) was added, and the mixture was vortexed and incubated at 80 °C for 30 min. Finally, 2 mL 0.9 % (wt/v) sodium chloride (NaCl) and 1 mL hexane were added, followed by vortexing and centrifugation at 2000×*g* for 2 min. The top hexane layer was then transferred to GC vials for analysis by gas chromatography-mass spectrometry (GC-MS). During GC-MS analysis, the temperature was initially held at 50 °C for 2 min, ramped to 200 °C at a rate of 25 °C/min, held for 1 min, and then raised to 315 °C at a rate of 25 °C/min. Helium was used as the carrier gas at a flow rate of 1 mL/min through a DB-5MS separation column (30 m, 0.25 mm ID, 0.25 μm, Agilent).

### 2.6 Cellular hydrophobicity analysis

Cells grown in the presence of 5 mM octanoic acid were harvested at mid-log phase. The cells were then centrifuged at 5000×*g* for 20 min, washed twice with 0.1 M PBS buffer, and resuspended to an optical density at 550 nm (OD_550_) of approximately 0.6. The true OD_550_ was determined using a spectrophotometer (A1). To extract the hydrocarbon-adherent cells, 1.2 mL of the cell suspension was transferred to 2.4 mL of hexane. The mixture was vortexed for 1 min and then left at room temperature for 15 min to allow for phase separation. Afterward, the lower aqueous phase was carefully removed, and the OD_550_ of this phase was measured (A2). The microbial adhesion to hydrocarbons (MATH) value was calculated using the following formula: MTAH = (A1 − A2)/A1.

### 2.7 Membrane ATP content characterization

Membrane intracellular ATP content was measured using an ATP assay kit (Beyotime) according to the manufacturer's instructions. Strains were cultured in MOPS medium, cells at logarithmic phase when OD_550_ = 2.0 were collected and weighted for calculation.

### 2.8 Statistical analysis

The statistical significance of all the data in this study was analyzed using the two-tailed *t*-test method. A P-value of less than 0.05 was statistically significant.

## 3 Results and discussion

### 3.1 Mining heterologous enzyme candidates for lanosterol biosynthesis in *E. coli*

**Fig. 1:**
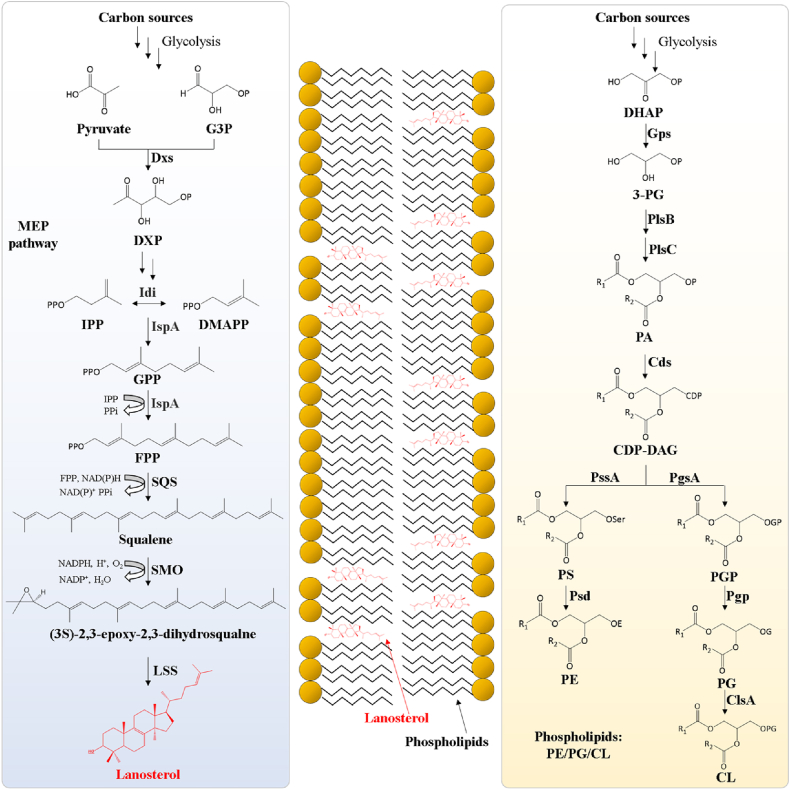
Overall schematic of lanosterol biosynthesis (left) and phospholipids biosynthesis (right) in *E. coli*. MEP, 2C-methyl-d-erythritol-4-phosphate; G3P, glyceraldehyde 3-phosphate; DXP, 1-deoxy-d-xylulose-50phosphate; IPP, isopentenyl pyrophosphate; DMAPP, dimethylallyl pyrophosphate; GPP, geranyl diphosphate; FPP, farnesyl pyrophosphate; Dxs, 1-deoxy-d-xylulose-5-phosphate; Idi, isopentenyl diphosphate isomerase; IspA, Farnesyl diphosphate synthase; SQS, squalene synthase; SMO, Squalene monooxygenase; LSS, Lanosterol synthase; DHAP, dihydroxyacetone phosphate; 3-PG, glycerol-3-phosphate; PA, phosphatidic acid; CDP-DAG, cytidine diphosphate diacylglycerol; PS, phosphatidylserine; PE, phosphatidylethanolamine; PGP, phosphatidylglycerophosphate; PG, phosphatidylglycerol; CL, cardiolipin; Gps, glycerol-3-phosphate dehydrogenase; PlsB, glycerol-3-phosphate acyltransferase; PlsC, 1-acylglycerol-3-phosphate O-acyltransferase; Cds, CDP-diglyceride synthetase; PssA, phosphatidylserine synthase; Psd, phosphatidylserine decarboxylase; PgsA, phosphatidylglycerophosphate synthase; Pgp, phosphatidylglycerophosphatase; ClsA, cardiolipin synthase.

Since lanosterol plays a crucial role as a precursor metabolite in the biosynthesis of sterols [8,9], we specifically utilized lanosterol to represent the broader category of sterols. In this regard, we aim to reconstruct the synthetic pathway of lanosterol in *E. coli* [24]. The native methylerythritol phosphate (MEP) pathway can provide the precursor farnesyl pyrophosphate (FPP) [15]. Through the introduction of the artificial heterologous pathway, we can realize the *de novo* synthesis of sterols in a bacterial model, *E. coli*. The heterologous pathway contains three exogenous enzymes, in which the first two enzymes tErg9 (encoding squalene synthase) and SMO_*Mc*_ (encoding squalene monooxygenase) had been mined in our previous work [15], and the third exogenous enzyme, lanosterol synthase (LSS) can cyclize the intermediate to produce the desired end-product, lanosterol (Fig. 1) [13].

For the search of LSS enzyme, we also selected candidates from the representative eukaryote *Saccharomyces cerevisiae* as our starting point., the gene *erg7* encodes the enzyme lanosterol synthase (LSS) [25]. After conducted the prokaryotic expression system, we found that ERG7 also exhibited poor solubility in *E. coli* (Fig. S1A). Inspired by the case of SMO_*Mc*_, we pursued the LSS candidate from *M. capsulatus* for further testing [26]. This candidate (LSS_*Mc*_) shares a 33 % primary sequence identity with ERG7 (Fig. S2). However, unlike SMO_*Mc*_, LSS_*Mc*_ displayed poor solubility in *E. coli* (Fig. S1B), indicating that additional efforts are needed to improve its solubility.

Affinity fusion tags have been proven to be effective tools for enhancing the solubility of heterologous proteins [27]. Therefore, we employed eight different fusion tags, including glutathione-S-transferase (GST), maltose binding protein (MBP), HaloTag (Halo), SUMO, Flag, N-utilization substance A (NusA), thioredoxin (TrxA), and guanine nucleotide-binding protein (GB1) [28]. To streamline the process and minimize trial and error, we utilized the Protein-Sol tool to predict the solubility of these fusion proteins [29]. The predicted solubility value of LSS_*Mc*_ alone was only 0.099, significantly lower than the average solubility value of soluble proteins in *E. coli* (∼0.45) [29], which was consistent with our initial experimental results (Fig. S1B). We then placed these eight tags at the N-terminal of LSS_*Mc*_ predicted by the Protein-Sol tool, resulting in eight different combinations (Table 1). Among these combinations, commonly used tags such as GST, MBP, and HaloTag showed limited improvement in the solubility of LSS_*Mc*_, with all scaled solubility values falling below 0.12. Interestingly, we found that the NusA tag greatly enhanced the solubility of LSS_*Mc*_, with the solubility value of NusA-LSS_*Mc*_ reaching 0.373, representing a 3.76-fold increase compared to LSS_*Mc*_ alone. To validate this observation, we experimentally expressed the NusA-LSS_*Mc*_ fusion protein in *E. coli* BL21(DE3) and confirmed a significant increase in its solubility (Fig. S1C). Therefore, we selected the NusA-LSS_*Mc*_ fusion protein as our candidate.

**Table 1:** Predicted solubility values of LSS_*Mc*_ with different tags.

Tag	Predicted solubility
No tag	0.099
+GST	0.059
+MBP	0.119
+Halo	0.105
+Sumo	0.113
+Flag	0.132
+**NusA**	**0.373**
+TrxA	0.088
+GB1	0.106

### 3.2 Introduction and optimization of lanosterol biosynthesis pathway in *E. coli*

**Fig. 2:**
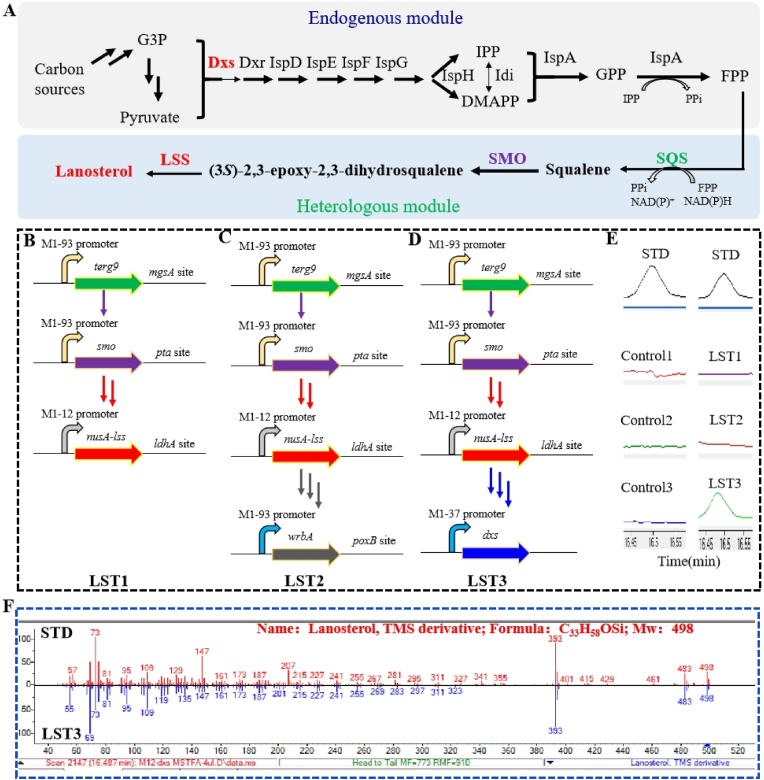
Introduction and optimization of lanosterol biosynthesis pathway in *E. coli.***A**, The full biosynthetic pathway of lanosterol consists of endogenous and heterologous modules; **B**, Integration of heterologous *S. cerevisiae terg9* gene, *M. capsulatus smo* gene with M1-93 artificial promoter into genomic DNA of *E. coli* MG1655 at the *mgsA* site and *pta* site, respectively. The *M. capsulatus lss* gene fused with NusA tag was integrated into genomic DNA of *E. coli* MG1655 at *ldhA* site with artificial promoter M1-12; **C**, based on b, *wrbA* gene with M1-93 artificial promoter was integrated into genomic DNA of *E. coli* MG1655 at *poxB* site. **D**, based on b, the native promoter of *dxs* was replaced with artificial promoter M1-37. **E–F**, GC-MS detection of lanosterol for the three engineered strains. The LST3 strain with the heterologous module and promoter replacement of rate-limiting enzyme *dxs* synthesized the representative lanosterol TMS derivative while control strain and other two engineered strains not.

After identifying potential enzymes for lanosterol synthesis, our next objective was to introduce three key genes into *E. coli* K-12 MG1655 (Fig. 2A). Used the previously engineered SOS1 strain [15] (MG1655 mgsA*:*:M1-93-*terg9*, *pta*::M1-93-*smo*_*Mc*_) as the original strain, we inserted the M1-12-*nusA*-*lss*_*Mc*_ expression cassettes at the *ldhA* site (Fig. 2b) [30]. This resulted in the creation of the engineered strain LST1 (MG1655 mgsA*::*M1-93-*terg9*, *pta::*M1-93-*smo*_*Mc*_, *ldhA::*M1-12-*nusA*-*lss*_*Mc*_).

However, we did not detect the presence of lanosterol or other sterols in the lipids of LST1 strain (Fig. 2E). Since SMO is a P450 monooxygenase that requires NADPH-dependent cytochrome P450 reductase (CPR) for catalysis [31], we speculated that the lack of approximate CPRs led to the failure in detecting lanosterol. To address this issue, we overexpressed a previously-reported *E. coli* endogenous CPR-like protein called NADH: quinone oxidoreductase (WrbA) [32]. By inserting an additional copy of the *wrbA* gene with the constitutive M1-93 promoter (M1-93-*wrbA*) at the *poxB* site in the LST1 strain, we obtained the LST2 strain (MG1655 mgsA*::*M1-93-*terg9*, *pta::*M1-93-*smo*_*Mc*_, *ldhA::*M1-12-*nusA*-*lss*_*Mc*_, *poxB::*M1-93-*wrbA*) (Fig. 2C). However, even with this *wrbA* overexpression, we still did not observe the production of lanosterol, indicating the existence of other bottlenecks in lanosterol biosynthesis (Fig. 2E).

The MEP pathway is responsible for converting various carbon sources into DMAPP and IPP precursors, but its regulation is tightly controlled in *E. coli* [33]. We hypothesized that further activation of the MEP pathway would enhance lanosterol biosynthesis. To achieve this, we up-regulated the expression of the key enzyme 1-deoxy-d-xylulose-5-phosphate synthase (Dxs), which has been identified as the rate-limiting step of the MEP pathway [38]. Specifically, we replaced the native promoter of the *dxs* gene with a strong-strength constitutive promoter M1-37. Finally, we obtained the engineered *E. coli* strain LST3 (MG1655 mgsA*::*M1-93-*terg9*, *pta::*M1-93-*smo*_*Mc*_, *ldhA::*M1-12-*nusA*-*lss*_*Mc*_, M1-37-*dxs*) (Fig. 2D). Similar manipulations were performed to create the control strain (MG1655 Δ*mgsA*, Δ*pta*, Δ*ldhA*, M1-37-*dxs*). Gas chromatography (GC) analysis successfully confirmed the presence of lanosterol in the cell lipids fraction of the LST3 strain but not in the control strain (Fig. 2E), full range GC spectra was shown in Fig. S3. The identity of the lanosterol produced was further verified using mass spectrometry (*m/z* of TMS derivative = 498) (Fig. 2F). Quantitative analysis revealed that the lanosterol content in the engineered *E. coli* strain was approximately 27 μg/g dry cell weight (DCW), although very low compare to the lanosterol content in eukaryotes which can reach to 0.5–1 mg/g [34], to the best of our knowledge, this represents the first *E. coli* strain containing lanosterol. In addition, no *(S)*-2,3-oxidosqualene intermediate production or other sterols, such as cholesterol and ergosterol, were detected in the LST3 strain.

### 3.3 Effects of lanosterol on tolerance of *E. coli*

**Fig. 3:**
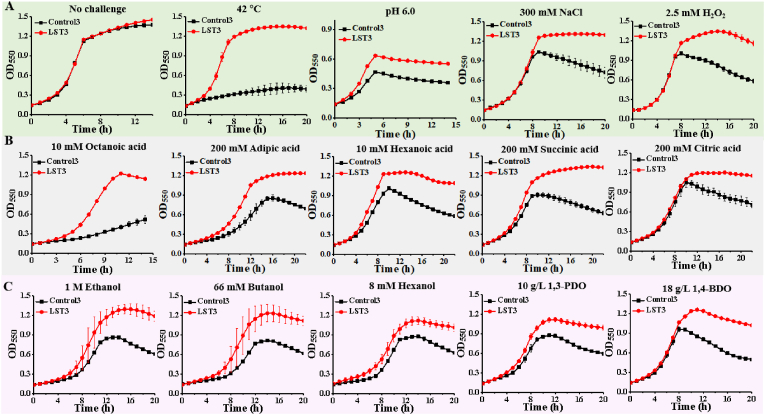
LST3 improves tolerance to adverse industrial conditions and a broad range of toxic chemicals. **A**, LST3 improves tolerance to high temperature, acidic condition, high osmotic pressure and ROS. **B**, LST3 improves tolerance to a broad range of organic acids. **C**, LST3 improves tolerance to alcohols. Growth curves were recorded in MOPS + 2 % glucose medium with different inhibitors in clear bottom 96-well plate at 37 °C (except for high temperature) with an initial pH of 7.0 (except for acidic condition). Values are the average of three biological replicates with error bars indicating one standard deviation.

Under standard cultivation conditions, such as 37 °C in MOPS + 2 % (wt/v) glucose minimal medium, the engineered LST3 strain showed no significant difference compared to the control strain. The specific growth rate (μ) of LST3 strain (0.333 h^−1^) was comparable to that of the control strain (0.341 h^−1^) (P > 0.05) (Fig. 3A). We further investigated whether the tolerance of LST3 increased. Intriguingly, when cultivated at a high temperature of 42 °C, LST3 exhibited a 3-fold increase in its μ compared to the control strain (0.376 h^−1^ vs 0.091 h^−1^, P = 0.01) (Fig. 3A). Furthermore, it was observed that the introduction of lanosterol allowed the engineered strain to shift its optimal growth temperature from 37 °C to 42 °C (0.333 h^−1^ vs 0.376 h^−1^). Aside from heat tolerance, LST3 also displayed improved tolerance to acidic conditions. Specifically, under an acidic condition with an initial pH of approximately 6.0, LST3 achieved its highest cell mass at OD_550_ ∼ 0.62, which was a 38 % improvement compared to the control strain (OD_550_ ∼ 0.45, P = 0.01) (Fig. 3A).

We further investigated the effect of lanosterol introduction on high osmotic pressure and reactive oxygen species (ROS). The results showed that the LST3 strain exhibited better growth than the control strain under 300 mM NaCl and 2.5 mM H_2_O_2_, both with higher maximum OD_550_ values (Fig. 3A). In addition to adverse conditions, lanosterol also enabled *E. coli* strain enhanced tolerance to a wide range of toxic chemicals, including organic acids. Specifically, the LST3 strain showed improved tolerance to octanoic acid (10 mM), with its μ increasing by 2.7-fold compared to the control strain (0.333 h^−1^ vs 0.091 h^−1^, P = 0.01) (Fig. 3B). This similar phenomenon was also observed when the strains were exposed to other organic acids such as adipic acid, hexanoic acid, succinic acid, and citric acid (Fig. 3B). In addition to organic acids, lanosterol also enhanced *E. coli*'s tolerance to alcohols (Fig. 3C). Monohydric alcohol and dihydric alcohol were selected for testing, and the results showed that LST3 exhibited an increased μ compared to the control strain in the presence of monohydric alcohol (Fig. 3C). Regarding dihydric alcohol, two representative diols were chosen for analysis: 1,3-propanediol (1,3-PDO) and 1,4-butanediol (1,4-BDO), as they are important precursors for polymer synthesis [35]. The continuous dynamic growth experiment results showed that the LST3 strain had a higher maximum OD_550_ than the control strain. In the presence of 10 g/L 1,3-PDO, the OD_550_ of LST3 was 1.12, whereas the control strain only reached 0.82, representing a 37 % increase. Similarly, in the presence of 18 g/L 1,4-BDO, the OD_550_ of LST3 was 1.23, whereas the control strain only reached 0.94, indicating a 31 % increase (Fig. 3C). Both the resistance analyses for organic acids and alcohols demonstrated the performance advantage of LST3 in terms of growth rate or biomass.

Based on the tolerance analysis results of LST3, which showed increased resistance to stress conditions comparing to its control cells. We further tested the broad-spectrum tolerance of LST1 and LST2 strains which are important controls to claim lanosterol is responsible for the phenotype. The results indicated that the tolerance of LST1 was comparable to that of the control (Fig. S4). In contrast, LST2 exhibited a slight improvement in tolerance to alcohols and pH 6.0 (Fig. S5). We hypothesize that this improvement maybe attributed to the overexpression of WrbA (a NADPH-dependent cytochrome P450 reductase). The overexpression of WrbA could enhance tolerance through multiple mechanisms, including anti-oxidation, maintaining the metabolic balance of NADH, regulating the global stress response, and collaborating with chaperone proteins. Overall, when comparing LST1, LST2 and LST3, the key differences were that LST2 had an overexpression of WrbA, while lanosterol was detected in LST3. Given that LST3 showed a more significant improvement in tolerance, it is reasonable to speculate that this enhancement can be ascribed to the presence of lanosterol.

### 3.4 Potential mechanism of the robustness of the engineered bacterium

**Fig. 4:**
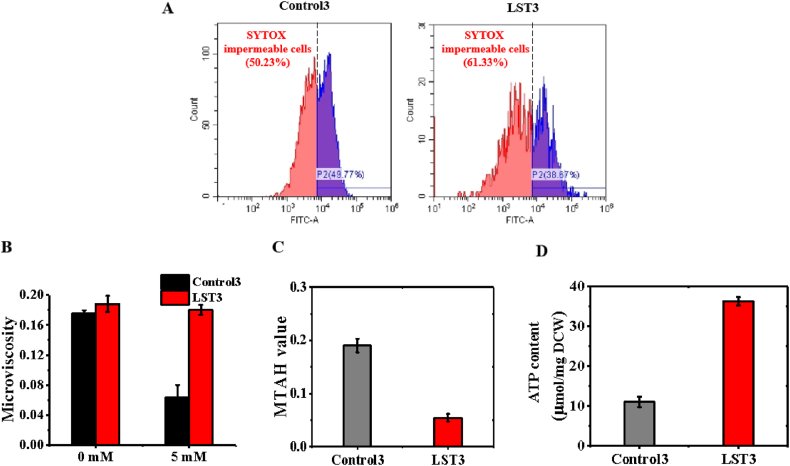
Contributions of increased membrane integrity, decreased fluidity, and decreased hydrophobicity, and higher ATP content to the improved tolerance of the LST3 strain. **A**, the LST3 strain exhibited increased cell membrane integrity, resulting in more impermeable membrane cells during the challenge of 5 mM octanoic acid. **B**, the LST3 strain-maintained cell membrane fluidity during the challenge of 5 mM octanoic acid, while the control strain could not. **C**, the LST3 strain exhibited decreased hydrophobicity when exposed to 5 mM octanoic acid. MTAH, microbial adhesion to hydrocarbons. **D**, the LST3 strain demonstrated a 3.3-fold in ATP content as compared to the control strain during the challenge of 5 mM octanoic acid. Values are the average of three biological replicates with error bars indicating one standard deviation.

Subsequently, our investigation focused on unraveling the potential underlying mechanism responsible for the enhanced tolerance observed in the LST3 strain. Previous studies have identified membrane leakage as a primary detrimental mechanism during cellular stress challenges [21,36,37]. Therefore, we first assessed the extent of membrane leakage in both the control and LST3 strains. To do this, we utilized octanoic acid, a representative membrane-damaging chemical [22,30], and added it to the cultures. Additionally, we also added the SYTOX green nucleic acid stain to facilitate the identification of cells with leaked cell membranes [21]. Our findings revealed that, when exposed to 5 mM octanoic acid, the proportion of SYTOX-impermeable cells in the LST3 strain comprised approximately 61 % of the population, which represented an increase of 22 % (P < 0.05) compared to the control strain's proportion of 50 % (Fig. 4A). Based on these results, we deemed that lanosterol led to a significant reduction in cell membrane leakage in the *E. coli* strain.

Furthermore, we measured another crucial membrane property, membrane fluidity, using the membrane polarization fluorescent probe 1,6-Diphenyl-1,3,5-hexatriene (DPH) [30], which binds to the membrane bilayer. In the absence of octanoic acid, the microviscosity (reciprocal value of membrane fluidity) of the LST3 strain (0.188 ± 0.01) showed no significant difference compared to the control strain (0.175 ± 0.004, P > 0.05) (Fig. 4B). However, when exposed to 5 mM octanoic acid, the microviscosity value of the control strain significantly decreased to 0.064 ± 0.016 (P < 0.01). This reduction is consistent with previous reports which demonstrated that the absence of octanoic acid increased membrane fluidity [38,39]. Interestingly, the LST3 strain maintained an almost identical microviscosity value (0.180 ± 0.007) under the challenge of 5 mM octanoic acid (Fig. 4B) as the no challenge condition, suggesting that lanosterol contributes to maintaining membrane fluidity.

Furthermore, we found that the LST3 strain exhibited decreased hydrophobicity relative to the control strain when treated with 5 mM octanoic acid, as indicated by the microbial adhesion to hydrocarbons (MATH) value (Fig. 4C). This observation aligns with previous studies demonstrating that lower surface hydrophobicity confers improved tolerance to organic solvents in *E. coli* [40]. Next, we continued to investigate the intracellular ATP content after introducing lanosterol into *E. coli*, as ATP serves as a versatile energy compound that powers various fitness-maintaining processes in living cells, and in bacteria, ATP synthase lies across the cell membrane [41]. During the challenge with 5 mM octanoic acid, the logarithmic phase strains were collected and weighted for the ATP content analysis, we observed that the average ATP content in the LST3 strain was 36.3 μmol/mg DCW, which represents a 3.3-fold (P = 0.001) as compared to the control strain (11.1 μmol/mg DCW) (Fig. 4D). In summary, we propose that the maintenance of membrane integrity, fluidity, reduced hydrophobicity, and higher ATP production collectively contribute to the enhanced tolerance observed in the LST3 strain.

### 3.5 Characterization analysis of the cell membrane

**Fig. 5:**
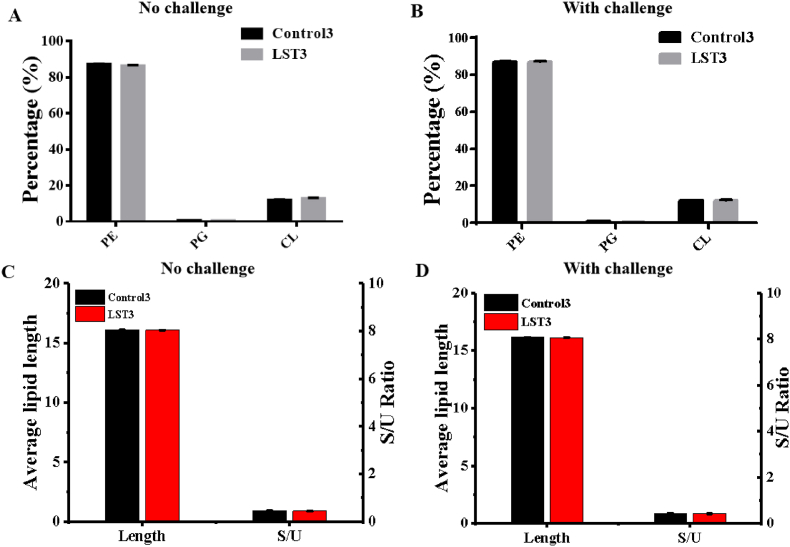
Characterization analysis of the cell membrane. **A–B**, proportion analysis of PE/PG/CL in the membrane lipids of the control strain and LST3 strain cultured in 0 mM (A) and 5 mM (B) octanoic acid MOPS medium. **C–D**, average lipid length and saturated/unsaturated ratio of fatty acid lipids in the control strain and LST3 strain cultured in 0 mM (C) and 5 mM (D) octanoic acid MOPS medium. Values are the average of three biological replicates with error bars indicating one standard deviation.

We subsequently conducted a comprehensive analysis of the cell membrane. Our objective was to investigate whether the introduction of the heterologous sterol biosynthesis pathway would impact phospholipid biosynthesis. In *E. coli*, the three native phospholipids (Fig. 1) are phosphatidylethanolamine (PE), phosphatidylglycerol (PG), and cardiolipin (CL) [42]. Previous studies have demonstrated that altering the distribution of native phospholipids can enhance cellular tolerance to various stresses [43]. However, in our study, we discovered that the distribution of these three phospholipids did not significantly change between the engineered LST3 strain and the control strain, both under normal conditions and when exposed to a representative challenge (e.g., 5 mM octanoic acid) (P > 0.05) (Fig. 5A and B). Furthermore, the average phospholipids length in the LST3 strain (16.14 ± 0.02) was also nearly identical to that of the control strain (16.12 ± 0.01), both in the absence or presence of 5 mM octanoic acid (Fig. 5C and D). Besides, the saturated/unsaturated (S/U) ratio of phospholipids in the LST3 strain (0.89 ± 0.0007) did not exhibit a significant difference compared to the control strain (0.91 ± 0.002) (P = 0.13) (Fig. 5C and D). Therefore, we inferred that this improved tolerance was achieved in a manner independent of phospholipids, and the introduction of lanosterol biosynthesis did not affect the biosynthesis of native phospholipids, indicating lanosterol biosynthesis has high orthogonality with phospholipid biosynthesis.

## 4 Conclusion

In this study, we have successfully reconstituted the biosynthesis of the eukaryotic lipid lanosterol in *E. coli.* The successful synthesis of lanosterol provides a novel platform for investigating lipid biosynthesis pathways related to eukaryogenesis and their functional implications. By introducing heterologous enzymes from *S. cerevisiae* and *M. capsulatus* [25,26], we demonstrated the ability of bacteria to produce sterols. Additionally, in the future, as more evidence can confirm that lanosterol is integrated into cell membrane, it will offer a unique opportunity to study the coexistence of heterologous and native lipids as a hybrid heterochiral membrane in living bacterial cells.

As we found that the introduction of lanosterol biosynthesis did not affect the biosynthesis of native phospholipids, so it's impact on membrane fluidity, integrity, and ATP production is closely related to its ability to modulate the biophysical properties of the cell membrane, though based on the current experimental results, the relationship between these factors is not clear. We hypothesize that lanosterol may contribute to the maintenance of membrane stability and function by modulating membrane fluidity. This regulatory effect could indirectly impact the functionality of the mitochondrial membrane, consequently influencing ATP synthesis. For instance, a slight decrease in membrane fluidity could potentially reinforce mitochondrial membrane stability, leading to enhanced ATP synthase activity [44]. Moreover, lanosterol's role in enhancing ATP production could be linked to its effects on membrane-associated ATPases. In eukaryotic systems, sterols like ergosterol and cholesterol are known to modulate the activity of H^+^-ATPase and other membrane-bound enzymes [7]. By analogy, lanosterol in the *E. coli* may similarly support efficient ATP synthesis, which is essential for maintaining cellular energy homeostasis and supporting stress response mechanisms. As for membrane proteins, a critical factor influencing the activity of them is membrane fluidity, including those involved in nutrient uptake, ion transport, and signal transduction. By reducing fluidity, lanosterol may stabilize membrane proteins in their functional conformations, thereby enhancing overall cellular robustness [7,45]. This stabilization is particularly important under stress conditions, such as high temperature, low pH, and exposure to ROS.

The tolerance mechanism was performed under the challenge of the most representative octanoic acid, which is widely accepted that the mechanism of bacterial tolerance improvement is relate to changes in membrane functions, as our research shown. We speculate that the effects of lanosterol on membrane properties and strain tolerance may vary under different stress conditions, for example, under high temperature, lanosterol may also participate in antioxidant defense, induction of heat shock protein expression, and regulation of plant hormone signaling pathways. In the future, it will be interesting to explore the action mechanism of lanosterol under different stress environments. The presence of lanosterol in the engineered *E. coli* strain confers enhanced tolerance to a range of adverse conditions frequently encountered in industrial environments. These include high temperature, low pH, ROS, and high osmotic pressure, all of which can significantly impact process efficiency and costs [15]. For example, by improving thermotolerance, the requirement for cooling water could be reduced, consequently lowering the likelihood of contamination risks and operational costs in industrial applications. Additionally, lanosterol enables the engineered strain to tolerate diverse range of toxic chemicals, such as organic acids and alcohols, making it a superior chassis for biotechnological applications compared to naturally existing *E. coli.*

The development of the *E. coli* LST3 strain will facilitate further studies on the compatibility of eukaryotic and bacterial lipids and their functional interactions. Understanding these interactions is crucial for elucidating the mechanisms by which lanosterol and other sterols influence membrane protein function, stress tolerance, and overall cellular physiology. We anticipate this study will stimulate further research into the biotechnological applications of engineered lipid systems as well as provide insights into the early evolutionary origins of sterol biosynthesis during cellular life evolution. By leveraging the unique properties of lanosterol and its derivatives, future work may focus on optimizing industrial microbial strains for enhanced productivity and robustness, as well as exploring the potential of hybrid lipid systems in synthetic biology and bioengineering.

## CRediT authorship contribution statement

**Wenjie Sun:** Methodology, Investigation. **Yun Chen:** Methodology, Investigation. **Syed Bilal Shah:** Methodology, Investigation. **Yanfen Bai:** Methodology, Investigation. **Zaigao Tan:** Writing – original draft, Supervision, Project administration, Methodology, Investigation, Conceptualization.

## Data availability

The data that support the findings of this study are available in the supplementary material of this article.

## Declaration of competing interest

The authors declare that they have no competing financial interests or personal relationships that could have appeared to influence the work reported in this paper.
