# Supplementary material for: Biosynthesis of lanosterol in Escherichia coli
Source: Synth Syst Biotechnol. 2025 May 13;10(3):993–1001. doi: 10.1016/j.synbio.2025.05.006 (PMC12146001; doi:10.1016/j.synbio.2025.05.006)
Supplement: Multimedia component 1 [file mmc1.docx]

Table S1. Strains and plasmids used in this study.

| Strains/plasmids | Characteristics | Source | |
| --- | --- | --- | --- |
| Strains |  |  |  |
| MG1655 | Wild-type *E. coli* K1-12 strain | Lab collection | |
| BL21(DE3) | F-ompT hsdSB (rB- mB-) gal dcm (DE3)  (prokaryotic protein expression system) | Vazyme | |
| Control 1 | MG1655, Δ*mgsA*, Δ*pta*, Δ*ldhA*, | This study | |
| Control 2 | MG1655, Δ*mgsA*, Δ*pta*, Δ*ldhA*, Δ*poxB* |  | |
| Control 3 | MG1655, Δ*mgsA,* Δ*pta*, Δ*ldhA*, M1-37-*dxs* | This study | |
| LST1 | MG1655, Δ*mgsA*::M1-93-*tErg9*, Δ*pta*::M1-93-*smo*, Δ*ldhA*::M1-12-NusA-*lss* | This study | |
| LST2 | MG1655, Δ*mgsA*::M1-93-*tErg9*, Δ*pta*::M1-93-*smo*, Δ*ldhA*::M1-12-NusA-*lss*, Δ*poxB*::M1-93-*wrbA*, | This study | |
| LST3 | MG1655, Δ*mgsA*::M1-93-*tErg9*, Δ*pta*::M1-93-*smo*, Δ*ldhA*::M1-12-NusA-*lss*, M1-37-*dxs* | This study | |
| Plasmids |  |  | |
| pCDF-duet1 | Carries the CloDF13 replicon, lacI gene and streptomycin/spectinomycin resistance gene (aadA). | Lab collection | |
| pCaS | Contains the *cas9* gene with a native promoter, an arabinose-inducible sgRNA guiding Cas9 to the *pMB1* replicon of pTarget, the λ-Red recombination system to improve the editing efficiency, and the temperature-sensitive replication *repA101*(Ts) for self-curing. | Lab collection | |
| pTargetF | Be constructed to express the targeting sgRNA | Lab collection | |
| pSJ7 | Used for NusA-tag cloning | Lab collection | |

Table S2. Primers and sequence used in this study.

| Name | Sequence | Use |
| --- | --- | --- |
| MgsA-L-up | CAACACGCTGGCCGAAGT | Used for amplification of MgsA left homologous arm |
| MgsA-L-down | ATCCAGTCGCCGCATTTC |  |
| MgsA-L-KO-down | CTGATGAGCTGGGTGGAACGATCCAGTCGCCGCATTTCAA | Used with MgsA-L-up for amplification of mgsA left homologous arm for mgsA knock out |
| MgsA-R-up | CGTTCCACCCAGCTCATC | Used for amplification of mgsA right homologous arm |
| MgsA-R-down | CTCGCCATTACCTCAACG |  |
| M1-93-tErg9-up | TTGAAATGCGGCGACTGGATTTATCTCTGGCGGTGTTG | Used for amplification of M1-93 promoter that fused to tErg9 |
| M1-93-tErg9-down | AATTGTAATAGCTTTCCCATAGCTGTTTCCTGGTTTAAAC |  |
| tErg9-up | ATGGGAAAGCTATTACAATTGGC | Used for amplification fragment of tErg9 that fused to mgsA-R |
| tErg9-Cas9-dn | CTGATGAGCTGGGTGGAACGTCAGTACTCTTCTTCTTGTTGGG |  |
| MgsA-L-up1 | TCACATGAGGCCTGCCAG | Used with mgsA-L-up1 for verification of tErg9 insertion |
| tErg9-YZ-down | GCAGCTCTCTGATCACAGC |  |
| pta-L-up | TGACCAAAGAGTCTGGCCT | Used for amplification of pta left homologous arm |
| pta-L-down | GGTTTATCCTCTTTCGTTACCG |  |
| pta-L-KO-down | AGCTGCGGATGATGACGAGAGGTTTATCCTCTTTCGTTACCG | Used with pta-L-up for amplification of pta left homologous arm for pta knock out |
| pta-R-up | TCTCGTCATCATCCGCAG | Used for amplification of pta right homologous arm |
| pta-R-down | GTCGTGAACAGCTGTACGC |  |
| M1-93-SMO-up | GTAACGAAAGAGGATAAACCTTATCTCTGGCGGTGTTG | Used for amplification of M1-93 promoter that fused to tErg9 |
| M1-93-SMO-down | CCCAGTTCGATGCTGCTCATAGCTGTTTCCTGGTTTAAAC |  |
| SMO-up | ATGAGCAGCATCGAACTGGG | Used for amplification fragment of smo that fused to pta-R |
| SMO-Cas9-dn | CTGCGGATGATGACGAGATTATTTAGCACGCAGCGCC |  |
| pta-L-up1 | TGAGCGTTGACGCAATCA | Used with pta-L-up1 for verification of SMO insertion |
| SMO-YZ-down | GTCCGGTTCAACGATCAGAA |  |
| ldhA-L-up | AAGGCTGGCGTTGGTGAT | Used for amplification of ldhA left homologous arm |
| ldhA-L-down | AAGACTTTCTCCAGTGATGTTG |  |
| ldhA-L-KO-down | AATGCAGGGGAGCGGCAAGAAAGACTTTCTCCAGTGATGTTG | Used with ldhA-L-up for amplification of ldhA left homologous arm for ldhA knock out |
| ldhA-R-up | TCTTGCCGCTCCCCTGC | Used for amplification of ldhA right homologous arm |
| ldhA-R-down | CGTTAACTGGTTCGCGGTC |  |
| M1-93-lss-up | ACATCACTGGAGAAAGTCTTTTATCTCTGGCGGTGTTG | Used for amplification of M1-12 promoter that fused to lss |
| M1-93-lss-down | AGGCTCAGCAGGTGTTTCATAGCTGTTTCCTGGTTTAAAC |  |
| lss-up | ATGAAACACCTGCTGAGCCT | Used for amplification of lss that fused to ldhA-R |
| lss-Cas-down | GCAGGGGAGCGGCAAGATTAACGACGGTAACCGGTAT |  |
| ldhA-L-up1 | CAAGCAGAATCAAGTTCTACCG | Used with ldhA-L-up1 for verification of lss insertion |
| lss-YZ-down | CAGCTGAGACAGTTCGAAAT |  |
| Lss-pSJ7-up | GCGACTAGTGGTTCTGGTATGAAACACCTGCTGAGCCT | Used for amplification of lss fragment constructed to pSJ7 vector |
| Lss-pSJ7-down | GAGTGATGGTGATGGTGATGTTAACGACGGTAACCGGTAT |  |
| pSJ7-F | CATCACCATCACCATCACTC | Used for amplification of pSJ7 backbone for lss fragment |
| pSJ7-R | ACCAGAACCACTAGTCGC |  |
| NusA-up | ATGAACAAAGAAATTTTGGCTGTAGT | Cloning for NusA-lss fused fragment |
| Lss-down | ATGAACAAAGAAATTTTGGCTGTAGT |  |
| poxB-L-up | GCGGCCCGGCTCCGTATATG | Used for amplification of poxB left homologous arm |
| poxB-L-down | GGTTCTCCATCTCCTGAATGTGATA |  |
| poxB-KO-down | GACGGGAAATGCCACCCTTTGGTTCTCCATCTCCTGAATGTGATA | Used with poxB-L-up for amplification of poxB left homologous arm for poxB knock out |
| poxB-R-up | AAAGGGTGGCATTTCCCGTCATAAT | Used for amplification of poxB right homologous arm |
| poxB-R-down | AATTCCCATGCTTCTTTCAG |  |
| M1-93-wrbA-up | TATCACATTCAGGAGATGGAGAACCTTATCTCTGGCGGTGTTGAC | Used for amplification of M1-93 promoter that fused to wrbA |
| M1-93wrbA-down | ATTATGACGGGAAATGCCACCCTTTttaGCCGTTAAGTTTAACTG |  |
| WrbA-up | atgGCTAAAGTTCTGGTGCT | Used for amplification fragment of wrbA that fused to poxB-R |
| WrbA-cas-down | ATTATGACGGGAAATGCCACCCTTTttaGCCGTTAAGTTTAACTG |  |
| poxB-L-up1 | CGAACTGCTGGCAGGTGTAC | Used with poxB-L-up1 for verification of wrbA insertion |
| WrbA-yz-down | CCGGACATGTTGCCAAAGCG |  |
| pTargetF-up | ACTAGTATTATACCTAGGACTGAG |  |
| pTargetF-mgsA-dn | GTCCTAGGTATAATACTAGTAACGTCAACGCGATGTTGAGGTTTTAGAGCTAGAAATAGC | Used with pTargetF-up to amplify pTargetF-mgsA |
| pTargetF-pta-dn | GTCCTAGGTATAATACTAGTGCTGATTCCGCTGCGGCCTTGTTTTAGAGCTAGAAATAGC | Used with pTargetF-up to amplify pTargetF-pta |
| pTargetF-ldhA-dn | GTCCTAGGTATAATACTAGTCTGACCGGCTTTACTATGTAGTTTTAGAGCTAGAAATAGC | Used with pTargetF-up to amplify pTargetF-ldhA |
| Dxs-M1-37-up | ACTACATCATCCAGCGTAATAAATAAACAATAAGTATTAATAGGCCCCTGAGGAACACTTAACGGCTGAC | Used for the amplification fragment of Dxs-M1-37-FRT-kana-FRT in the one-step recombination |
| Dxs-M1-37-down | GTGGAGTCGACCAGTGCCAGGGTCGGGTATTTGGCAATATCAAAACTCATAGCTGTTTCCTGGTTTAAAC |  |


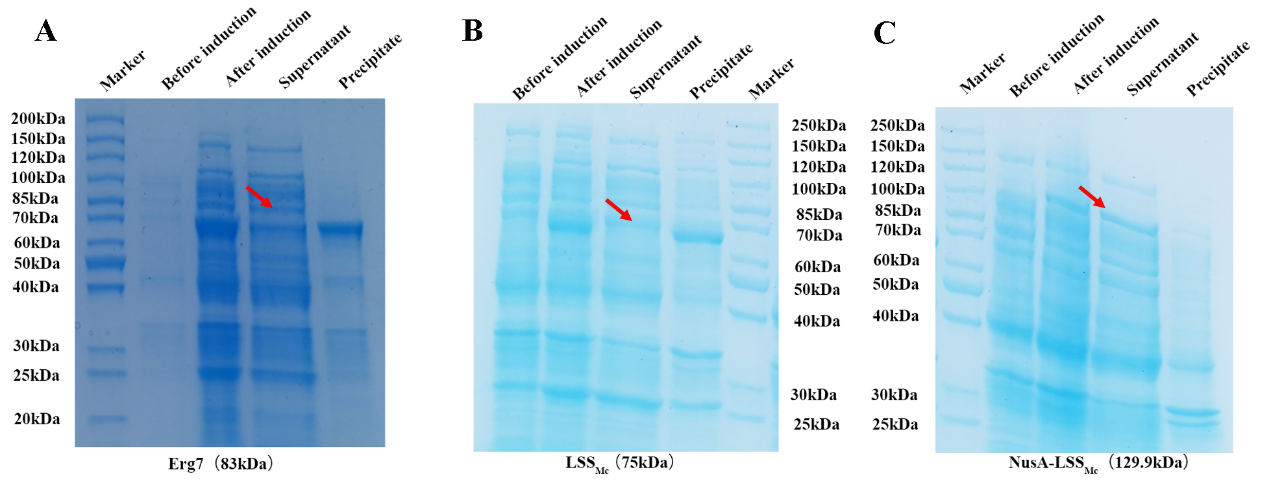


Figure S1. Mining enzyme candidates for lanosterol biosynthesis in *E. coli* using the prokaryotic expression system. **A**, Erg7, lanosterol synthase from *S. cerevisiae*; **B**, LSS*_Mc_*, lanosterol synthase from *M. capsulatus*; **C**, NusA-LSS*_Mc_*, the affinity fusion tag NusA was fusioned with LSS*_Mc_*.


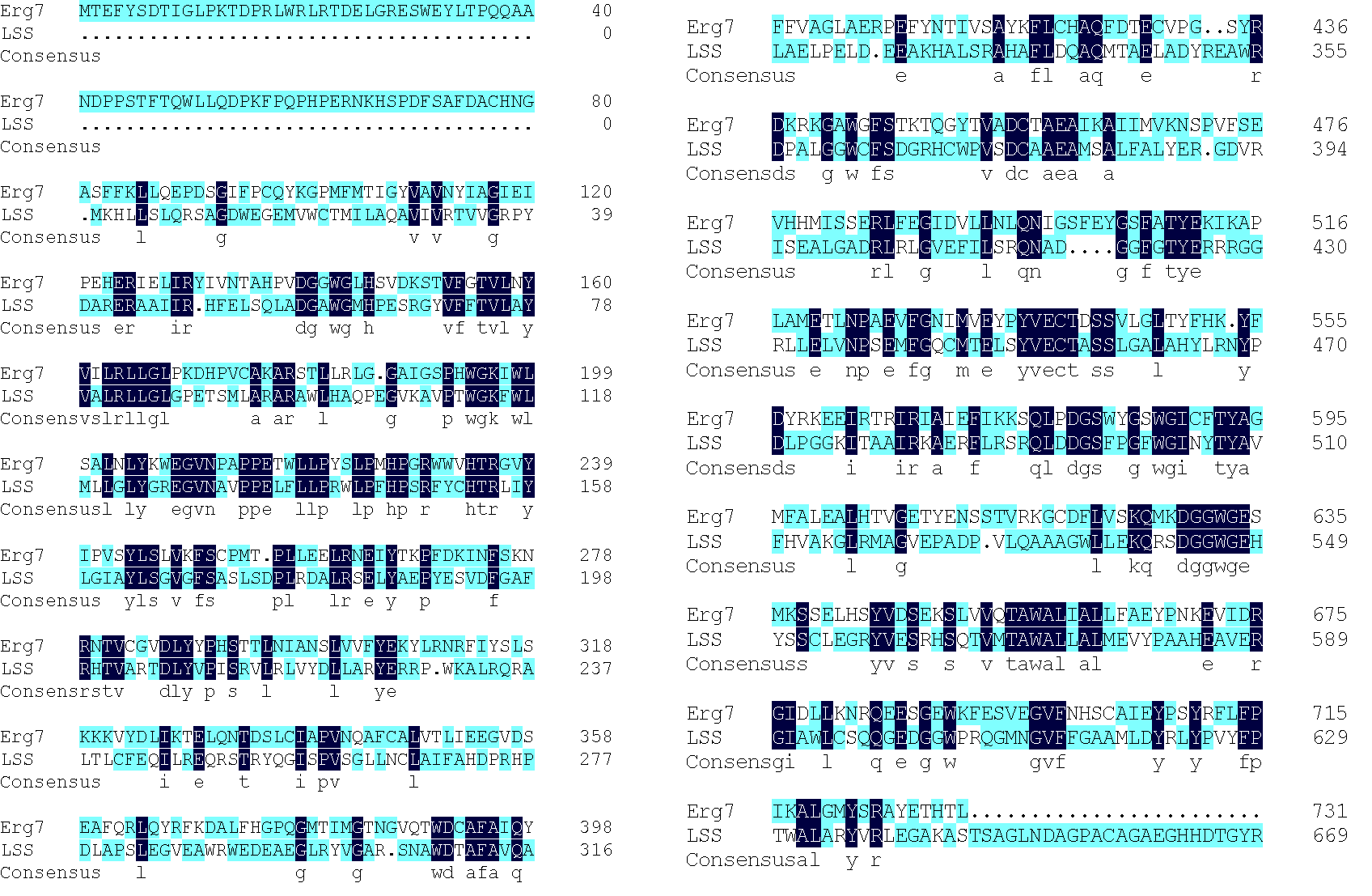


Figure S2. LSS from *Methylococcus capsulatus* shares a 33% primary protein sequence identity with ERG7 from *Saccharomyces cerevisiae.*


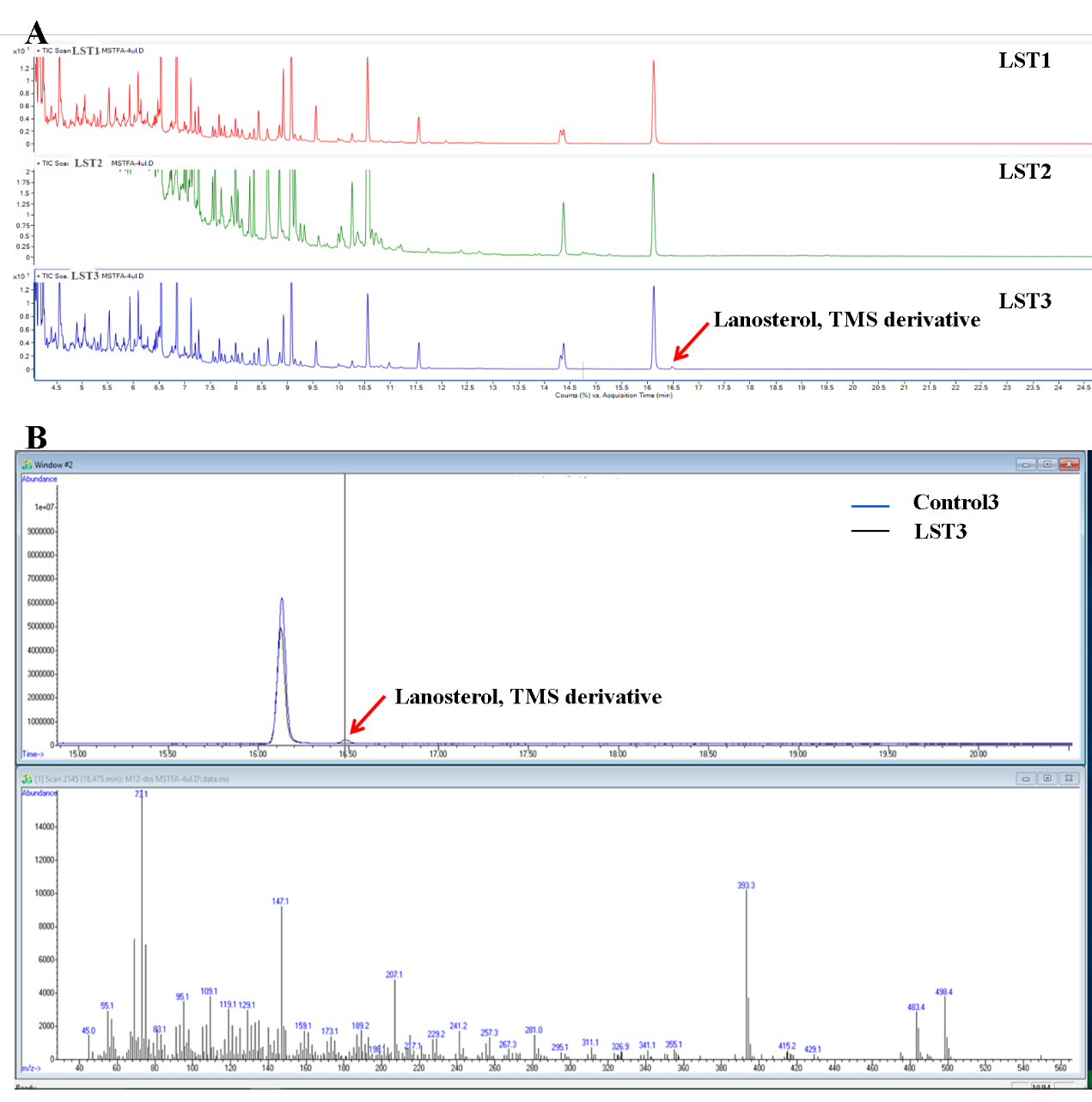


Fig. S3: full range GC spectra of LST1、LST2、LST3 (A), as well as the overlap of LST3 with its control strain (B).


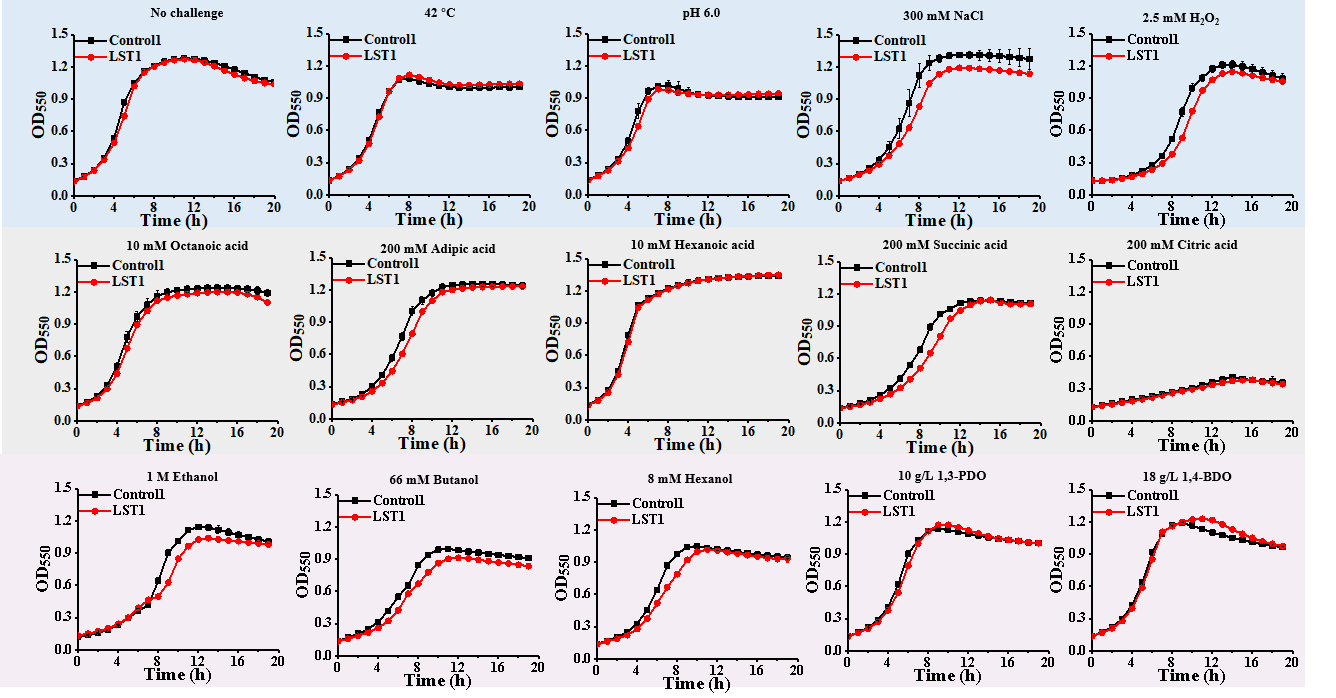


Fig. S4: The broad-spectrum tolerance analysis of LST1 compare to its control strain


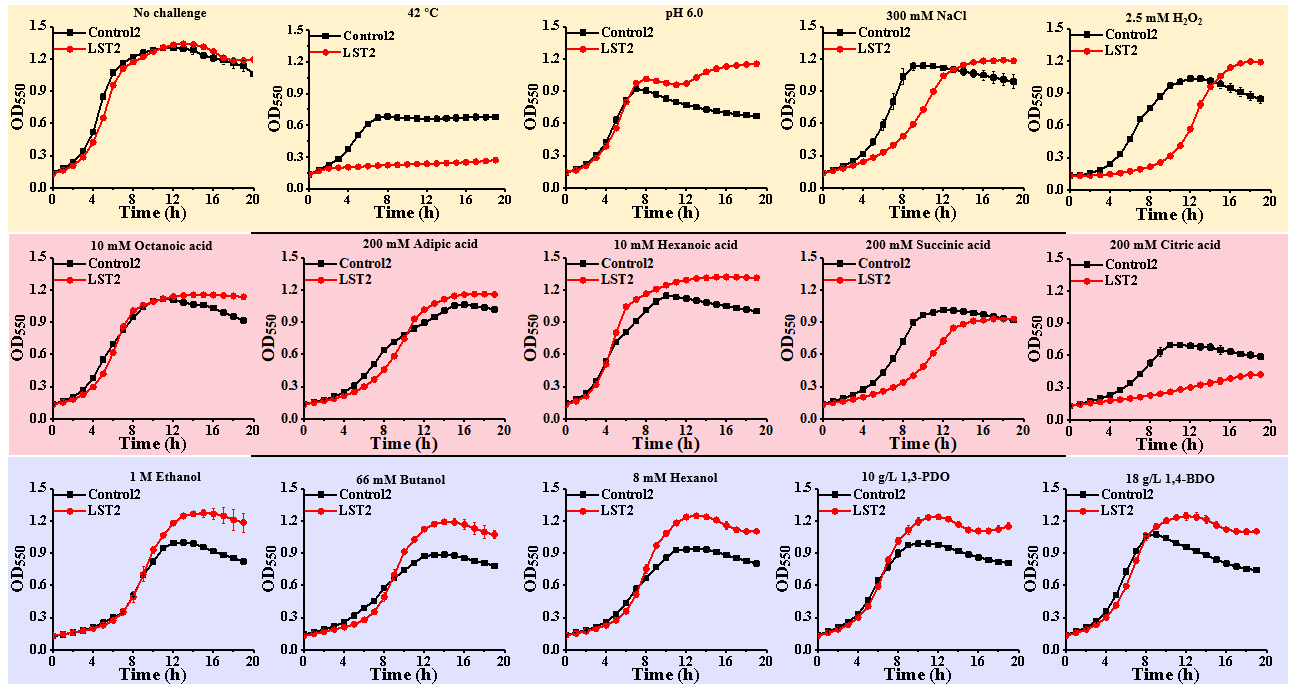


Fig. S5: The broad-spectrum tolerance analysis of LST2 compare to its control strain
